# Resveratrol Ameliorates the Depressive-Like Behaviors and Metabolic Abnormalities Induced by Chronic Corticosterone Injection

**DOI:** 10.3390/molecules21101341

**Published:** 2016-10-13

**Authors:** Yu-Cheng Li, Ya-Min Liu, Ji-Duo Shen, Jun-Jie Chen, Yang-Yi Pei, Xiao-Yan Fang

**Affiliations:** College of Pharmacy, Henan University of Traditional Chinese Medicine, Zhengzhou 450046, China; yamin62261@163.com (Y.-M.L.); lycdd1219@163.com (J.-D.S.); junjie102493@163.com (J.-J.C.); 18203977548@163.com (Y.-Y.P.)

**Keywords:** resveratrol, corticosterone, depression, metabolic abnormalities

## Abstract

Chronic glucocorticoid exposure is known to cause depression and metabolic disorders. It is critical to improve abnormal metabolic status as well as depressive-like behaviors in patients with long-term glucocorticoid therapy. This study aimed to investigate the effects of resveratrol on the depressive-like behaviors and metabolic abnormalities induced by chronic corticosterone injection. Male ICR mice were administrated corticosterone (40 mg/kg) by subcutaneous injection for three weeks. Resveratrol (50 and 100 mg/kg), fluoxetine (20 mg/kg) and pioglitazone (10 mg/kg) were given by oral gavage 30 min prior to corticosterone administration. The behavioral tests showed that resveratrol significantly reversed the depressive-like behaviors induced by corticosterone, including the reduced sucrose preference and increased immobility time in the forced swimming test. Moreover, resveratrol also increased the secretion of insulin, reduced serum level of glucose and improved blood lipid profiles in corticosterone-treated mice without affecting normal mice. However, fluoxetine only reverse depressive-like behaviors, and pioglitazone only prevent the dyslipidemia induced by corticosterone. Furthermore, resveratrol and pioglitazone decreased serum level of glucagon and corticosterone. The present results indicated that resveratrol can ameliorate depressive-like behaviors and metabolic abnormalities induced by corticosterone, which suggested that the multiple effects of resveratrol could be beneficial for patients with depression and/or metabolic syndrome associated with long-term glucocorticoid therapy.

## 1. Introduction

Glucocorticoid is an important hormone produced by adrenal cortex in response to the activation of hypothalamic-pituitary-adrenal (HPA) axis. An appropriate glucocorticoid level is necessary to maintain normal physiological functions. Elevated glucocorticoid levels can inhibit the activity of HPA axis through a feedback regulation mechanism [1]. In addition, glucocorticoid is also an important drug for treating severe infections, autoimmune diseases or Addison’s disease, etc. However, numerous side effects caused by chronic usage of glucocorticoid are uncomfortable. The main side effect of glucocorticoid is Cushing’s syndrome, which consists of a cluster of metabolic abnormalities, osteoporosis, and hypertension and so on. Interestingly, it shares many characteristics with metabolic syndrome, such as abdominal obesity, glucose intolerance and dyslipidemia [2,3]. These metabolic abnormalities strongly raised the risk of cardiovascular diseases [4,5].

Chronic excess glucocorticoid can impair the neurogenesis and synaptic plasticity in the hippocampus [6,7,8], which may contribute to the occurrence of depression. Previous studies have shown that an elevated blood glucocorticoid level is frequently found in patients with depression [9] and animal models induced by chronic mild stress [10,11]. In our and others’ previous studies, a mice model of depression had been successfully established by chronic corticosterone (CORT) injection [12,13,14]. Another study reported that long-term corticosterone exposure induced depressive-like behavior and insulin resistance [15]. In fact, depression is a common comorbidity of metabolic syndrome [16,17] or Cushing syndrome [18,19]. Therefore, it is critical to improve abnormal metabolic status as well as depressive-like behavior in patients with hypercortisolism or long-term glucocorticoid exposure.

**Figure 1:**
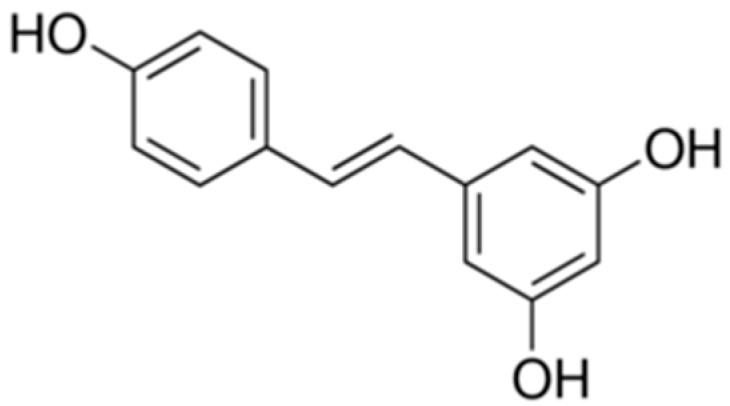
The structure of resveratrol.

Resveratrol is a naturally occurring polyphenolic compound that widely exists in various plants such as grapevines, peanuts and pomegranates (Figure 1). Resveratrol possesses numerous important pharmacological activities, including anti-oxidant, anti-diabetic, anti-inflammatory, anti-tumor, lipid-lowering properties and so on [20,21]. Recent studies have found antidepressant effects of resveratrol in animal models [22,23,24]. Ali et al have shown that resveratrol improved the depressive-like behavior induced by corticosterone [25]. However, whether resveratrol can improve the metabolic abnormalities induced by corticosterone remains unknown. Therefore, the present study aimed to investigate the effects of resveratrol on depressive-like behaviors and metabolic abnormalities induced by chronic corticosterone exposure. Our results will be beneficial for expanding the applications of resveratrol.

## 2. Results

### 2.1. Effects of Resveratrol on Body Weight

**Figure 2:**
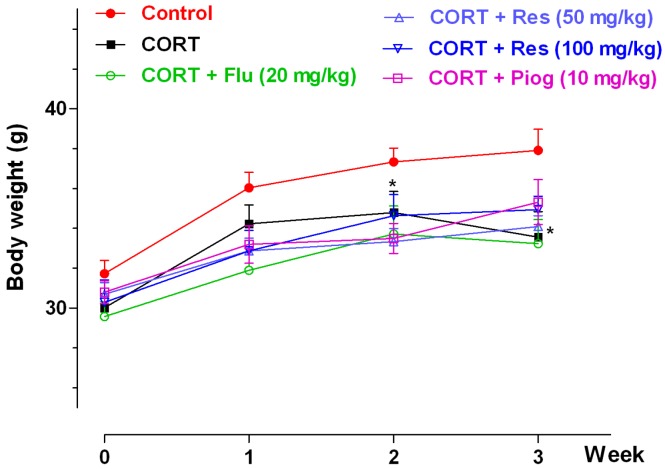
Effects of resveratrol on the body weight in mice. Data were expressed as the mean ± S.E.M. (*n* = 10). * *p* < 0.05 vs. control group. Data was analyzed by two-way repeated-ANOVA with week as the repeat factor. The difference between groups at the same time point was analyzed by Dunnett’s post hoc test. CORT: corticosterone; Flu: fluoxetine; Res: resveratrol; Piog: pioglitazone.

The effects of resveratrol on body weight were shown in Figure 2. In the control and CORT-vehicle groups, the repeated ANOVA showed a significant week effect (*p* < 0.001) and group effect (*p* < 0.001), but without an interaction effect. A post hoc test showed the body weight in CORT-vehicle group was significantly decreased at week 2 (*p* < 0.05) and week 3 (*p* < 0.05). In all CORT-treated groups, the repeated ANOVA showed a significant week effect (*p* < 0.001), but with non-significance of group and interaction effect. Pioglitazone, fluoxetine and resveratrol had no significant effect on body weight in the CORT-treated group.

### 2.2. Effects of Resveratrol on Depressive-Like Behaviors Induced by CORT

**Figure 3:**
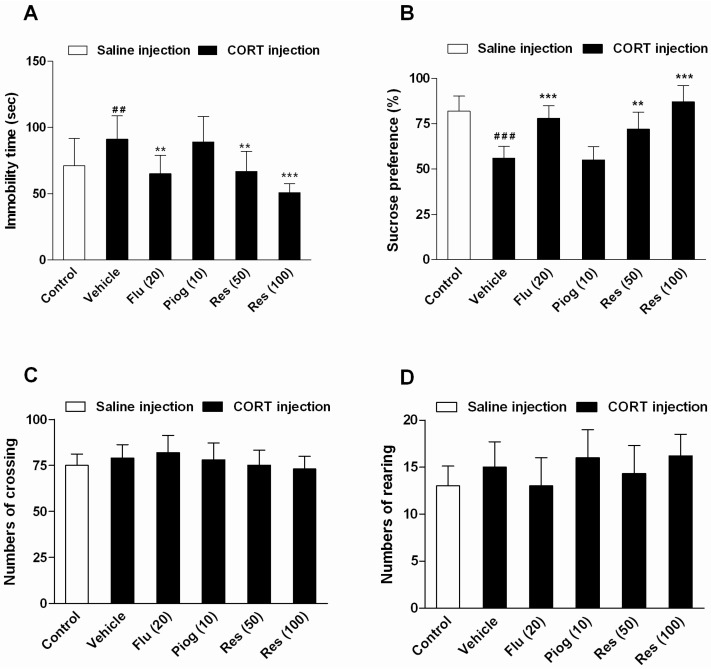
Effects of resveratrol on the depressive-like behaviors induced by CORT. (**A**) The immobility time in forced swimming test (FST); (**B**) The sucrose preference in sucrose preference test (SPT); (**C**) The numbers of crossing in open field test (OFT); (**D**) The numbers of rearing in OFT. Data were expressed as the mean ± S.E.M. (*n* = 10). ^##^ *p* < 0.01 and ^###^ *p* < 0.001 vs. control group. ** *p* < 0.01 and *** *p* < 0.001 vs. CORT-vehicle group. Data was analyzed by one-way ANOVA followed by Dunnett’s post hoc test.

As shown in Figure 3, chronic CORT exposure increased the immobility time in the forced swimming test (FST) (*p* < 0.01) and decreased the sucrose preference in the sucrose preference test (SPT) (*p* < 0.001). Resveratrol and fluoxetine significantly decreased the immobility time (Res: 50 mg/kg: *p* < 0.01; 100 mg/kg: *p* < 0.001; Flu: *p* < 0.01) and elevated the sucrose preference (Res: 50 mg/kg: *p* < 0.01; 100 mg/kg: *p* < 0.001; Flu: *p* < 0.001). However, pioglitazone had no significant effect on these behaviors. In addition, resveratrol, fluoxetine and pioglitazone didn’t affect the locomotor activity in open field test (OFT).

### 2.3. Effects of Resveratrol on the Insulin Sensitivity in CORT-Treated Mice

**Figure 4:**
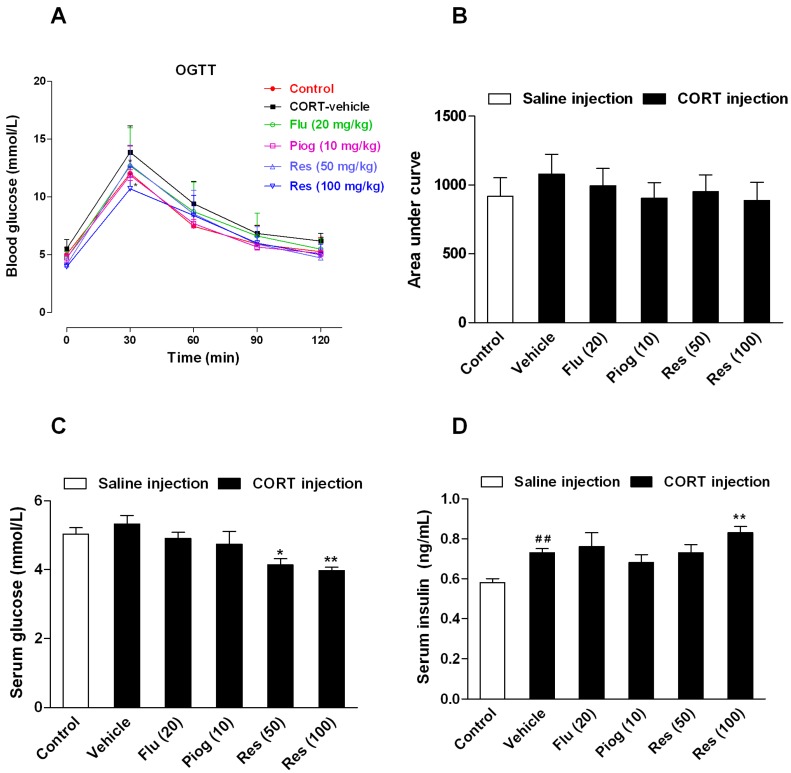
Effects of resveratrol on the insulin sensitivity in CORT-treated mice. (**A**) The curve of blood glucose level during OGTT; (**B**) The area under curve during OGTT; (**C**) The fasting serum glucose level; (**D**) The fasting serum insulin level. Data were expressed as the mean ± S.E.M. (*n* = 10). ^##^ *p* < 0.01 vs. control group. * *p* < 0.05, and ** *p* < 0.01 vs. CORT-vehicle group. Data of OGTT was analyzed by two-way repeated-ANOVA with time as the repeat factor. The data of AUC, glucose and insulin were analyzed by one-way ANOVA followed by Dunnett’s post hoc test.

Although the area under curve (AUC) during oral glucose tolerance test (OGTT) and the fasting serum glucose differences between control and CORT-vehicle group were non-significant, the fasting serum insulin levels were significantly increased following CORT exposure (*p* < 0.01). Resveratrol and pioglitazone slightly decreased the AUC during OGTT with no statistical significance. However, resveratrol significantly increased the fasting serum level of insulin (100 mg/kg: *p* < 0.01) and decreased the fasting serum level of glucose (50 mg/kg: *p* < 0.05; 100 mg/kg: *p* < 0.01). Pioglitazone and fluoxetine had no significant effect on these changes. These results are presented in Figure 4.

### 2.4. Effects of Resveratrol on the Serum Level of Lipids and Hormones in CORT-Treated Mice

**Figure 5:**
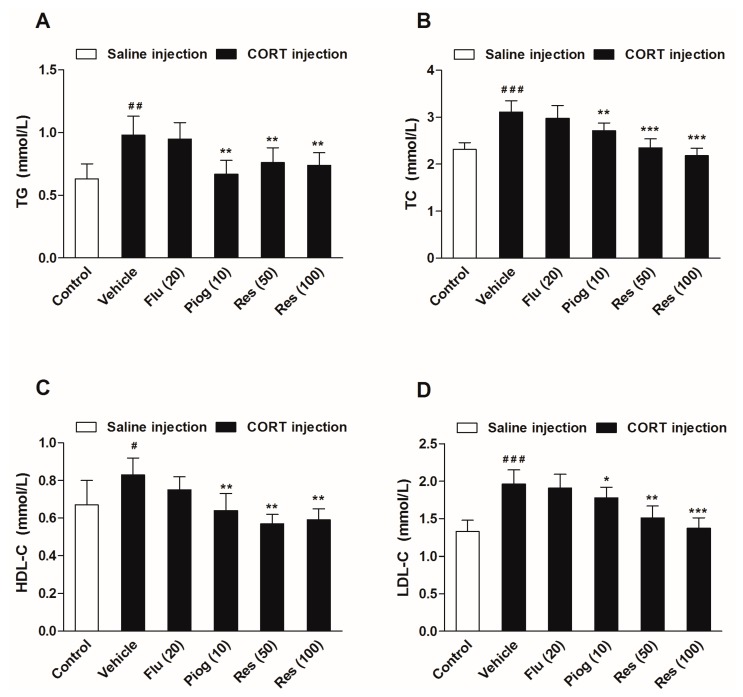
Effects of resveratrol on the serum lipid profiles in CORT- treated mice. (**A**) Serum level of TG; (**B**) Serum level of TC; (**C**) Serum level of HDL-C; (**D**) Serum level of LDL-C. Data were expressed as the mean ± S.E.M. (*n* = 10). ^#^ *p* < 0.05, ^##^ *p* < 0.01 and ^###^ *p* < 0.001 vs. control group. * *p* < 0.05, ** *p* < 0.01 and *** *p* < 0.001 vs. CORT-vehicle group. Data was analyzed by one-way ANOVA followed by Dunnett’s post hoc test. CORT: corticosterone; TG: triglycerides; TC: total cholesterol; HDL-C: high density lipoprotein cholesterol; LDL-C: low density lipoprotein cholesterol.

As displayed in Figure 5, chronic CORT exposure significantly increased serum levels of triglycerides (TG) (*p* < 0.01), total cholesterol (TC) (*p* < 0.001), high density lipoprotein cholesterol (HDL-C) (*p* < 0.05) and low density lipoprotein cholesterol (LDL-C) (*p* < 0.001). Resveratrol (50 and 100 mg/kg) reversed the alterations of TG (*p* < 0.01, *p* < 0.01), TC (*p* < 0.001, *p* < 0.001), HDL-C (*p* < 0.01, *p* < 0.01) and LDL-C (*p* < 0.01, *p* < 0.001) induced by CORT. Pioglitazone remarkably decreased TG (*p* < 0.01), TC (*p* < 0.01), HDL-C (*p* < 0.01) and LDL-C levels (*p* < 0.05) in CORT-treated mice. Fluoxetine had no significant effect on the serum lipids levels.

**Figure 6:**
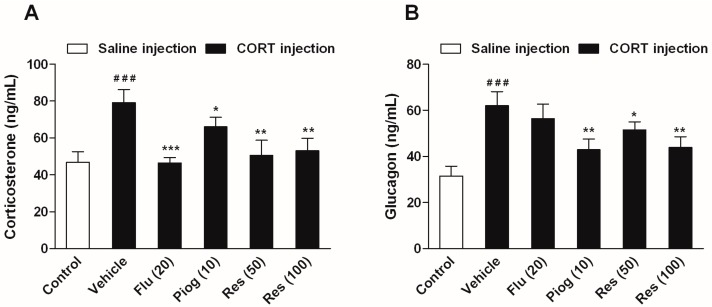
Effects of resveratrol on the serum hormone levels in CORT- treated mice. (**A**) Serum level of corticosterone; (**B**) Serum level of glucagon. Data were expressed as the mean ± S.E.M. (*n* = 10). ^###^ *p* < 0.001 vs. control group. * *p* < 0.05, ** *p* < 0.01 and *** *p* < 0.001 vs. CORT-vehicle group. Data was analyzed by one-way ANOVA followed by Dunnett’s post hoc test.

In addition, CORT also caused a significant elevation of serum corticosterone and glucagon levels (*p* < 0.001, *p* < 0.001). Both resveratrol and pioglitazone significantly reduced the serum corticosterone (Res: 50 mg/kg: *p* < 0.01; 100 mg/kg: *p* < 0.01; Piog: *p* < 0.05) and glucagon levels (Res: 50 mg/kg: *p* < 0.05; 100 mg/kg: *p* < 0.01; Piog: *p* < 0.01). Fluoxetine decreased the serum corticosterone level (*p* < 0.001), but didn’t affect the serum glucagon level. The results are shown in Figure 6.

### 2.5. Effects of Resveratrol on the Serum Biochemical Levels in Normal Mice

**Figure 7:**
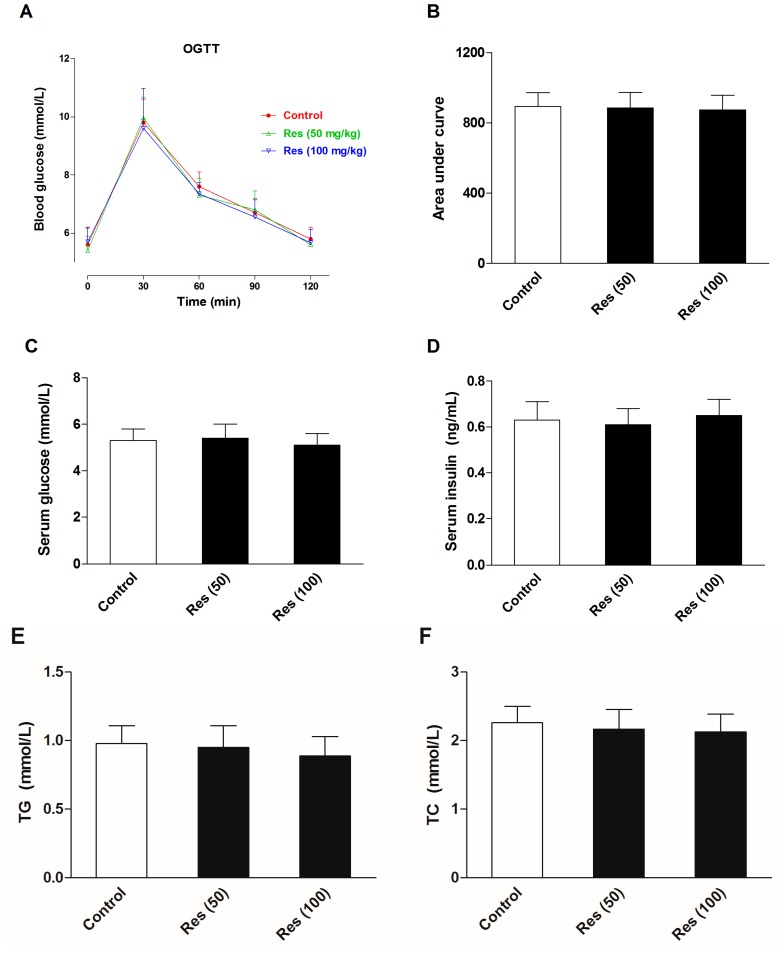

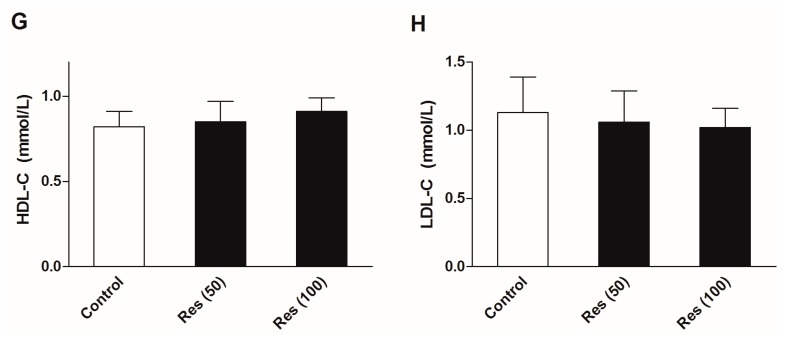
Effects of resveratrol on the serum biochemical levels in normal mice. (**A**) The curve of blood glucose level during OGTT; (**B**) The area under curve during OGTT; (**C**) The fasting serum glucose level; (**D**) The fasting serum insulin level; (**E**) Serum level of TG; (**F**) Serum level of TC; (**G**) Serum level of HDL-C; (**H**) Serum level of LDL-C. Data were expressed as the mean ± S.E.M. (*n* = 10). One-way ANOVA followed by Dunnett’s post hoc test were used for statistical analysis.

To evaluate the effects of resveratrol on the serum biochemical levels in normal mice, another batch of mice were employed in an additional experiments without corticosterone treatment. Thirty mice were divided into three groups. Except for the control group, groups were administrated 50 and 100 mg/kg resveratrol for 21 days. The OGTT was performed and serum biochemical levels were examined. All of the experimental methods were in agreement with the description in the methods section. The results are displayed in Figure 7. The results showed that resveratrol had no significant effects on the insulin sensitivity, serum levels of glucose and insulin, or blood lipid profiles in normal mice.

## 3. Discussion

The main finding of the present study is that resveratrol can reverse depressive-like behaviors, as well as metabolic abnormalities induced by chronic corticosterone exposure. Fluoxetine can only ameliorate depressive-like behaviors, while pioglitazone only improve metabolic abnormalities. These results suggested that the multiple effects of resveratrol could be beneficial for patients with combined disease.

Firstly, we found that corticosterone induced a significant reduction of sucrose preference and increased immobility time in the FST. It is well known that the decreased sucrose consumption and increased immobility time in FST are characterized as the core symptom of depression in rodents [26,27]. The present result showed that the mice model of depression had been successfully established by chronic corticosterone injection. The compounds which can reverse these behavioral changes will be further studied as the potent antidepressants. In the present study, resveratrol and fluoxetine dramatically reversed depressive-like behaviors induced by corticosterone without affecting locomotor activity. These results were agreement with the observations of Ali et al. [25]. Again, we verified the antidepressant effect of resveratrol in corticosterone-induced mice model of depression. However, it is different from the study of Ali et al. [25]. We chose resveratrol at dose of 50 and 100 mg/kg, while Ali et al. investigated the antidepressant effect of resveratrol at single dose of 80 mg/kg [25].

Besides depression, previous studies had confirmed that long-term glucocorticoid exposure caused metabolic abnormalities, including abdominal obesity, glucose intolerance, dyslipidemia and hypertension, etc. [2,28,29]. However, it should be noted that not all patients or animals received chronic glucocorticoids treatment represent all these metabolic abnormalities. Sometimes the results may be contradictory. For example, numerous studies have sustained that chronic glucocorticoid lead to an increased body weight [15,30], while others reported the opposite [14,31,32]. Notably, glucocorticoid is a catabolic hormone and is able to promote the decomposition of muscle and adipose tissue. Our study showed that chronic corticosterone injection significantly prevented the body weight gain. It could be ascribed to the accelerated decomposition and wastage of protein. This phenomenon had also been observed in rats undergo chronic dexamethasone treatment [33]. Another possible reason might be explained as different route of administration. Zhao et al., Lee et al. and our present study administrated corticosterone by subcutaneous injection [14,31], whereas Fransson et al. and Karatsoreos et al. treated with corticosterone via the drinking water and thus avoided the repeated stress of injections [28,30]. Resveratrol, fluoxetine and pioglitazone failed to reverse the alteration of body weight in the present study.

As we all known, insulin is an important hormone in maintaining metabolic homeostasis, including lowering blood glucose, promoting protein synthesis and inhibiting lipolysis, etc. It should, however, be noted that glucocorticoid can inhibit uptake and utilization of glucose in liver and skeletal muscle by interfering with insulin signaling and thereby leading to insulin resistance or glucose intolerance. In the study of Fransson et al, a 5-weeks corticosterone exposure dose- and time-dependently increased fasting serum insulin and C-peptide levels, but didn’t alter fasting blood glucose levels. Nevertheless, a clearly insulin resistance had been characterized by intraperitoneal insulin tolerance test (IPinsTT) and intraperitoneal glucose tolerance test (IPGTT) in corticosterone-treated mice [34]. Similarly, we also found a significant increase in fasting serum level of insulin, but no alteration in fasting serum level of glucose. The present results suggested that the β-cell had to compensate for the secretion of insulin to counteract high concentration of corticosterone and maintain blood glucose level. Fransson et al. also observed that corticosterone induced a significant increased β-cell proliferation and pancreatic islet volume, and thereby enhanced insulin secretion capacity. In addition, our results showed that the AUC during OGTT was slightly increased followed 3-weeks corticosterone exposure [34]. It could be explained as the 3-weeks corticosterone exposure was too transient to induce intensely insulin resistance. Or maybe, it could also be attributed to the fact that the administration of glucose by intraperitoneal injection avoids the incretin secretion.

Although resveratrol had no significant effect on the AUC during OGTT in the present study, it remarkably increased the fasting serum insulin and decreased fasting serum glucose level. This result suggested resveratrol were able to promote the secretion of insulin from pancreatic β-cell and strengthen the action of insulin. A great number of studies have illustrated that resveratrol could enhance pancreatic β-cell function by inhibiting phosphodiesterase activity [35], and reverse insulin resistance induced by lipopolysaccharides in mice [36] or endothelial cells induced by palmitate [37], improve hepatic insulin signaling in streptozotocin-induced diabetes rats [38]. Neither fluoxetine nor pioglitazone had effect on the fasting serum insulin and glucose level. Pioglitazone is one of the thiazolidines insulin sensitizer, but its capacity to reduce blood glucose is extremely weak. Our present study has also shown that repeated corticosterone injection induced a significant elevation of serum corticosterone and glucagon, which further indicated that the actions of insulin were severely disrupted in corticosterone treated mice. The levels of corticosterone and glucagon were significantly decreased followed resveratrol treatment, which indicated that resveratrol could be beneficial for improving insulin action and restoring metabolic homeostasis. In addition to promote gluconeogenesis and glycogenolysis, corticosterone can accelerate lipolysis and produce a large amount of TG and free fatty acids. The present results showed that corticosterone induced a significant increase in serum levels of TG, TC, HDL-C and LDL-C. These results also confirmed that chronic corticosterone exposure enhanced lipolysis activity and disarranged blood lipid profiles. These alterations were significantly reversed by resveratrol and pioglitazone treatment, but not fluoxetine. Moreover, we also found that resveratrol did not affect the serum levels of insulin, glucose and lipids in normal mice. These results further confirmed that the excellent protective effect of resveratrol against metabolic abnormilties induced by corticosterone, without affecting normal subjects.

## 4. Materials and Methods

### 4.1. Chemicals and Reagents

Resveratrol and corticosterone with purities of 98% were purchased from Sigma-Aldrich (St. Louis, MO, USA). Fluoxetine hydrochloride was from Changzhou Siyao Pharmaceuticals Co., Ltd. (Changzhou, China). Pioglitazone hydrochloride was from Jiangsu Deyuan Pharmaceuticals Co., Ltd. (Lianyungang, China). The triglycerides (TG), total cholesterol (TC), high density lipoprotein cholesterol (HDL-C), low density lipoprotein cholesterol (LDL-C) and glucose kits were purchased from Nanjing Jiancheng Bioengineering Institute (Nanjing, China). Insulin, corticosterone and glucagon ELISA kits were supplied by CUSABIO (Wuhan, China).

### 4.2. Animals

Male ICR mice weighing 18–22 g were procured from the Hunan Slac Animal Center, Changsha, China. The mice were housed ten per cage except for the sucrose preference test. The environment was maintained with a 12-h light/dark cycle (08:00–20:00). Ambient temperature and relative humidity were maintained at 22 ± 2 °C and at 40%–60%. The mice had free access to drink and food. The procedure was approved by the Committee of Animal care of Henan University of Traditional Chinese Medicine.

### 4.3. The CORT-Treated Procedure

**Figure 8:**
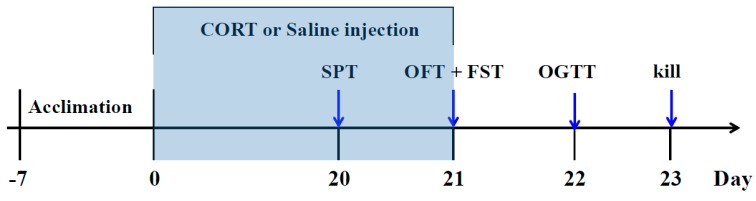
Schedule of the experiment.

After one week of adaptation, the mice were randomly divided into six groups (*n* = 10): (1) Control group: saline (p.o) + saline (s.c); (2) CORT-vehicle group: saline (p.o) + CORT (s.c); (3) Res group: resveratrol (50 mg/kg, p.o) + CORT (s.c); (4) Res group: resveratrol (100 mg/kg, p.o) + CORT (s.c); (5) Flu group: fluoxetine (20 mg/kg, p.o) + CORT (s.c); (6) Piog group: pioglitazone (10 mg/kg, p.o) + CORT (s.c). The dose of CORT was 40 mg/kg. The preparation of CORT solution was in line with our previous study [12]. In brief, the CORT was dissolved in a saline solution containing 0.1% dimethyl sulfoxide and 0.1% Tween-80. Resveratrol, fluoxetine and pioglitazone were given by oral gavage in a volume of 10 mL/kg 30 min prior to the CORT injection. The doses of resveratrol were chosen according to the previous reports [39,40]. The treatment was last for 21 days. The body weight was evaluated every week. The experimental schedule is displayed in Figure 8.

### 4.4. The Depressive-Like Behavior Test

The sucrose preference test (SPT) was performed at day 20. As described in our previous study [12], all mice were allowed to adapt to the individual cage and trained to consume sucrose solution (1% *w*/*v*) for 24 h without any water or food. Followed the sucrose intake training, the mice were deprived of food and water for 24 h, and then two bottles with 1% sucrose (100 mL) and tip water (100 mL) were randomly placed for another 24 h. The liquids consumption volume were measured and the sucrose preference was determined using the formula:

Sucrose preference (%) = [sucrose intake/(sucrose intake + water intake)] × 100%
(1)

Twenty-four hours after SPT, the forced swimming test (FST) was performed as described previously by Porsolt et al. with minor modifications [27]. In brief, the procedure was done by placing each mouse in a vertical glass cylinder (diameter 15 cm, height 30 cm) filled with 10 cm of water. The water temperature maintained at 23–25 °C. The mice were allowed to swim for 6 min, and the immobility time of last 4 min was recorded. The immobility period was considered as the time spent by mice floating in the water without struggling and making only those movements necessary to keep their head above the water.

To rule out the false positive FST results, the open field test (OFT) was performed 1 h prior to the FST. Without any interference, each mouse was individually placed in the center of a black box (40 cm in length, 40 cm in width, and 30 cm in height) with 25 equal squares (8 cm in length and width). The numbers of crossing and rearing were counted in 3-min session.

### 4.5. Oral Glucose Tolerance Test (OGTT)

The OGTT was conducted 24 h after FST. Followed a 14-h fasting, mice were administrated 50% glucose solution by oral gavage (1.5 g/kg), and then the blood glucose was determined at 0, 30, 60, 90, 120 min after administration by a Roche blood glucose meter.

### 4.6. Serum Biochemical Assay

After 24 h of OGTT, all mice were sacrificed by decapitation between 9:00–11:00 a.m. The serum was obtained by centrifuged at 4 °C, 1000 *g* for 10 min. The serum levels of glucose, TG, TC, HDL-C and LDL-C were detected using biochemical kits. The serum levels of insulin, corticosterone and glucagon were detected using ELISA kits.

### 4.7. Statistical Analysis

All data were expressed as mean ± S.E.M. The SPSS ver. 18.0 software (SPSS Inc., Chicago, IL, USA) was used for statistical analysis. The data of body weight at every week were analyzed by a two-way repeated-ANOVA with week as the repeat factor. The behavior data and biochemical data were analyzed by one-way ANOVA followed by Dunnett’s post hoc test. A value of *p* < 0.05 was considered as statistically significance.

## 5. Conclusions

In summary, the present results demonstrate that resveratrol could reverse the depressive-like behaviors and metabolic abnormalities induced by chronic corticosterone exposure. Based on these findings, we speculate that resveratrol may be beneficial for patients with depression and/or metabolic syndrome associated with long-term glucocorticoid therapy.

## References

[1] Boyle M.P., Kolber B.J., Vogt S.K., Wozniak D.F., Muglia L.J. (2006). Forebrain glucocorticoid receptors modulate anxiety-associated locomotor activation and adrenal responsiveness. J. Neurosci..

[2] Anagnostis P., Athyros V.G., Tziomalos K., Karagiannis A., Mikhailidis D.P. (2009). Clinical review: The pathogenetic role of cortisol in the metabolic syndrome: A hypothesis. J. Clin. Endocrinol. Metab..

[3] Kassi E., Pervanidou P., Kaltsas G., Chrousos G. (2011). Metabolic syndrome: Definitions and controversies. BMC Med..

[4] Després J.P. (2012). Body fat distribution and risk of cardiovascular disease: An update. Circulation.

[5] Liu A., Abbasi F., Reaven G.M. (2011). Adiposity indices in the prediction of metabolic abnormalities associated with cardiovascular disease in non-diabetic adults. Nutr. Metab. Cardiovasc. Dis..

[6] Anacker C., Cattaneo A., Luoni A., Musaelyan K., Zunszain P.A., Milanesi E., Rybka J., Berry A., Cirulli F., Thuret S. (2013). Glucocorticoid-related molecular signaling pathways regulating hippocampal neurogenesis. Neuropsychopharmacology.

[7] Gould E., Cameron H.A., Daniels D.C., Woolley C.S., McEwen B.S. (1992). Adrenal hormones suppress cell division in the adult rat dentate gyrus. J. Neurosci..

[8] Stranahan A.M., Arumugam T.V., Cutler R.G., Lee K., Egan J.M., Mattson M.P. (2008). Diabetes impairs hippocampal function through glucocorticoid-mediated effects on new and mature neurons. Nat. Neurosci..

[9] Pariante C.M. (2009). Risk factors for development of depression and psychosis. Glucocorticoid receptors and pituitary implications for treatment with antidepressant and glucocorticoids. Ann. N. Y. Acad. Sci..

[10] Li M., Fu Q., Li Y., Li S., Xue J., Ma S. (2014). Emodin opposes chronic unpredictable mild stress induced depressive-like behavior in mice by upregulating the levels of hippocampal glucocorticoid receptor and brain-derived neurotrophic factor. Fitoterapia.

[11] Yi L.T., Luo L., Wu Y.J., Liu B.B., Liu X.L., Geng D., Liu Q. (2015). Circadian variations in behaviors, BDNF and cell proliferation in depressive mice. Metab. Brain Dis..

[12] Li Y.C., Wang L.L., Pei Y.Y., Shen J.D., Li H.B., Wang B.Y., Bai M. (2015). Baicalin decreases SGK1 expression in the hippocampus and reverses depressive-like behaviors induced by corticosterone. Neuroscience.

[13] Wu T.C., Chen H.T., Chang H.Y., Yang C.Y., Hsiao M.C., Cheng M.L., Chen J.C. (2013). Mineralocorticoid receptor antagonist spironolactone prevents chronic corticosterone induced depressive-like behavior. Psychoneuroendocrinology.

[14] Zhao Y., Ma R., Shen J., Su H., Xing D., Du L. (2008). A mouse model of depression induced by repeated corticosterone injections. Eur. J. Pharmacol..

[15] Van Donkelaar E.L., Vaessen K.R., Pawluski J.L., Sierksma A.S., Blokland A., Cañete R., Steinbusch H.W. (2014). Long-term corticosterone exposure decreases insulin sensitivity and induces depressive-like behaviour in the C57BL/6NCrl mouse. PLoS ONE.

[16] Dunbar J.A., Reddy P., Davis-Lameloise N., Philpot B., Laatikainen T., Kilkkinen A., Bunker S.J., Best J.D., Vartiainen E., Lo S.K. (2008). Depression: An important comorbidity with metabolic syndrome in a general population. Diabetes Care.

[17] Skilton M.R., Moulin P., Terra J.L., Bonnet F. (2007). Associations between anxiety, depression, and the metabolic syndrome. Biol. Psychiatry.

[18] Bratek A., Koźmin-Burzyńska A., Górniak E., Krysta K. (2015). Psychiatric disorders associated with Cushing’s syndrome. Psychiatr. Danub..

[19] Kelly W.F., Checkley S.A., Bender D.A., Mashiter K. (1983). Cushing’s syndrome and depression—A prospective study of 26 patients. Br. J. Psychiatry.

[20] Saud S.M., Li W., Morris N.L., Matter M.S., Colburn N.H., Kim Y.S., Young M.R. (2014). Resveratrol prevents tumorigenesis in mouse model of Kras activated sporadic colorectal cancer by suppressing oncogenic Kras expression. Carcinogenesis.

[21] Yao J., Wang J.Y., Liu L., Li Y.X., Xun A.Y., Zeng W.S., Jia C.H., Wei X.X., Feng J.L., Zhao L. (2010). Anti-oxidant effects of resveratrol on mice with DSS-induced ulcerative colitis. Arch. Med. Res..

[22] Ge L., Liu L., Liu H., Liu S., Xue H., Wang X., Yuan L., Wang Z., Liu D. (2015). Resveratrol abrogates lipopolysaccharide-induced depressive-like behavior, neuro-inflammatory response, and CREB/BDNF signaling in mice. Eur. J. Pharmacol..

[23] Liu S., Li T., Liu H., Wang X., Bo S., Xie Y., Bai X., Wu L., Wang Z., Liu D. (2016). Resveratrol exerts antidepressant properties in the chronic unpredictable mild stress model through the regulation of oxidative stress and mTOR pathway in the rat hippocampus and prefrontal cortex. Behav. Brain. Res..

[24] Zhao X., Yu C., Wang C., Zhang J.F., Zhou W.H., Cui W.G., Ye F., Xu Y. (2014). Chronic resveratrol treatment exerts antihyperalgesic effect and corrects co-morbid depressive like behaviors in mice with mononeuropathy: Involvement of serotonergic system. Neuropharmacology.

[25] Ali S.H., Madhana R.M., Athira K.V., Kasala E.R., Bodduluru L.N., Pitta S., Mahareddy J.R., Lahkar M. (2015). Resveratrol ameliorates depressive-like behavior in repeated corticosterone-induced depression in mice. Steroids.

[26] Willner P., Towell A., Sampson D., Sophokleous S., Muscat R. (1987). Reduction of sucrose preference by chronic unpredictable mild stress, and its restoration by a tricyclic antidepressant. Psychopharmacology.

[27] Porsolt R.D., Bertin A., Jalfre M. (1977). Behavioral despair in mice: A primary screening test for antidepressants. Arch. Int. Pharmacodyn. Ther..

[28] Fransson L., Dos S.C., Wolbert P., Sjöholm A., Rafacho A., Ortsäter H. (2014). Liraglutide counteracts obesity and glucose intolerance in a mouse model of glucocorticoid-induced metabolic syndrome. Diabetol. Metab. Syndr..

[29] Gulliford M.C., Charlton J., Latinovic R. (2006). Risk of diabetes associated with prescribed glucocorticoids in a large population. Diabetes Care.

[30] Karatsoreos I.N., Bhagat S.M., Bowles N.P., Weil Z.M., Pfaff D.W., McEwen B.S. (2010). Endocrine and physiological changes in response to chronic corticosterone: A potential model of the metabolic syndrome in mouse. Endocrinology.

[31] Lee B., Sur B., Kwon S., Yeom M., Shim I., Lee H., Hahm D.H. (2013). Chronic administration of catechin decreases depression and anxiety-like behaviors in a rat model using chronic corticosterone injections. Biomol. Ther..

[32] Yu J., Yu B., He J., Zheng P., Mao X.B., Han G.Q., Chen D.W. (2014). Chronic glucocorticoid exposure-induced epididymal adiposity is associated with mitochondrial dysfunction in white adipose tissue of male C57BL/6J mice. PLoS ONE.

[33] Chimin P., Farias T.S.M., Torres-Leal F.L., Bolsoni-Lopes A., Campaña A.B., Andreotti S., Lima F.B. (2014). Chronic glucocorticoid treatment enhances lipogenic activity in visceral adipocytes of male Wistar rats. Acta Physiol..

[34] Fransson L., Franzén S., Rosengren V., Wolbert P., Sjöholm Å., Ortsäter H. (2013). β-Cell adaptation in a mouse model of glucocorticoid-induced metabolic syndrome. J. Endocrinol..

[35] Rouse M., Younès A., Egan J.M. (2014). Resveratrol and curcumin enhance pancreatic β-cell function by inhibiting phosphodiesterase activity. J. Endocrinol..

[36] Nøhr M.K., Dudele A., Poulsen M.M., Ebbesen L.H., Radko Y., Christensen L.P., Jessen N., Richelsen B., Lund S., Pedersen S.P. (2016). LPS-enhanced glucose-stimulated insulin secretion is normalized by resveratrol. PLoS ONE.

[37] Liu Z., Jiang C., Zhang J., Liu B., Du Q. (2016). Resveratrol inhibits inflammation and ameliorates insulin resistant endothelial dysfunction via regulation of AMP-activated protein kinase and sirtuin 1 activities. J. Diabetes.

[38] Sadi G., Pektaş M.B., Koca H.B., Tosun M., Koca T. (2015). Resveratrol improves hepatic insulin signaling and reduces the inflammatory response in streptozotocin-induced diabetes. Gene.

[39] Zheng X., Zhu S., Chang S., Cao Y., Dong J., Li J., Long R., Zhou Y. (2013). Protective effects of chronic resveratrol treatment on vascular inflammatory injury in streptozotocin-induced type 2 diabetic rats: Role of NF-κB signaling. Eur. J. Pharmacol..

[40] Haohao Z., Guijun Q., Juan Z., Wen K., Lulu C. (2015). Resveratrol improves high-fat diet induced insulin resistance by rebalancing subsarcolemmal mitochondrial oxidation and antioxidantion. J. Physiol. Biochem..

